# Coexisting choroidal neovascularization and active retinochoroiditis—an uncommon presentation of ocular toxoplasmosis

**DOI:** 10.1186/s12348-015-0051-2

**Published:** 2015-07-12

**Authors:** Sharat Hegde, Nidhi Relhan, Avinash Pathengay, Abhishek Bawdekar, Himadri Choudhury, Animesh Jindal, Harry W Flynn

**Affiliations:** GMRV Campus, LV Prasad Eye Institute, Visakhapatnam, India; Retina and Uveitis services, GMR Varalakshmi Campus, 11-113/1, Hanumantha waka Junction, Visakhapatnam, 530 040, Andhra Pradesh India

**Keywords:** Anti-vascular endothelial growth factor (anti-VEGF), Choroidal neovascular membrane, *Toxoplasma* retinochoroiditis

## Abstract

**Background:**

Choroidal neovascularization during the active stage of *Toxoplasma* retinochoroiditis is an uncommon clinical presentation. The authors retrospectively reviewed medical charts of patients with coexisting choroidal neovascular membrane and active *Toxoplasma* retinochoroiditis.

**Findings:**

Three patients presented with coexisting choroidal neovascular membrane and active *Toxoplasma* retinochoroiditis. All lesions had adjacent subretinal hemorrhage. The diagnosis was confirmed based on clinical presentation, fundus fluorescein angiography (FFA), and optical coherence tomography (OCT) findings. The patients were managed with a combination of treatments including intravitreal injection of anti-vascular endothelial growth factor (anti-VEGF), oral anti-*Toxoplasma* treatment, and oral corticosteroids. In all patients, the retinitis lesion resolved in 6 weeks and the coexisting choroidal neovascular membrane resolved over 6 to 12 weeks.

**Conclusions:**

Recurrences in *Toxoplasma* retinochoroiditis are common as satellite lesions adjacent to an old atrophic scar. Coexisting choroidal neovascularization with active *Toxoplasma* retinochoroiditis is an important presentation and should be suspected in the presence subretinal hemorrhage and managed with a combination of anti-*Toxoplasma* treatment and intravitreal anti-VEGF.

## Findings

### Background

Necrotizing retinochoroiditis caused by an obligate intracellular parasite, *Toxoplasma gondii*, is a common inflammatory lesion of the fundus accounting for up to 70 % of cases with retinochoroiditis [1, 2]. By virtue of high affinity for neural tissue and retinal ganglion cells [3], the *T. gondii* localizes in retina and causes recurring ocular inflammation. Focal necrotizing retinitis adjacent to old retinochoroidal scar is the characteristic lesion in ocular toxoplasmosis. The diagnosis of ocular toxoplasmosis can be made on the basis of clinical findings alone [3]. In the year 1969, Freidman and Knox [4] described the following three clinical presentations of active toxoplasmic retinochoroiditis (which occurs due to inflammatory response to activation of congenital toxoplasmosis [5]):

1. Large destructive active retinitis with associated vitritis (most common);
2. Punctate inner areas of retinitis with minimal associated edema and vitreous reaction;
3. Deep retinal punctate lesions with subretinal exudate (most unusual) associated with a minimal amount of vitreous reaction and with turbid subretinal fluid or blood.

When these lesions heal, they lead to scars with an atrophic, “punched out” appearance and variable pigmentary changes. The various reported late complications include secondary glaucoma, retinochoroidal vascular anastomosis, capillary non-perfusion, branch retinal artery and vein occlusion, choroidal neovascularization, cystoid macular edema, and optic atrophy [6]. Choroidal neovascularization (CNV) developing at the margins of the healed *Toxoplasma* scar lesion is an important cause of vision loss in young patients with maculopathy [2]. The prevalence of choroidal neovascular membrane (CNVM) in toxoplasmosis cases is reported to be 2–19 % [7, 8] during the late stage of the disease [9, 10]. CNV has been well reported to occur during the stage of healed toxoplasmosis [2, 9]. However, CNVM coexisting with active retinochoroiditis is uncommon. We report the clinical presentation and management of three such patients.

### Case 1

**Fig. 1:**
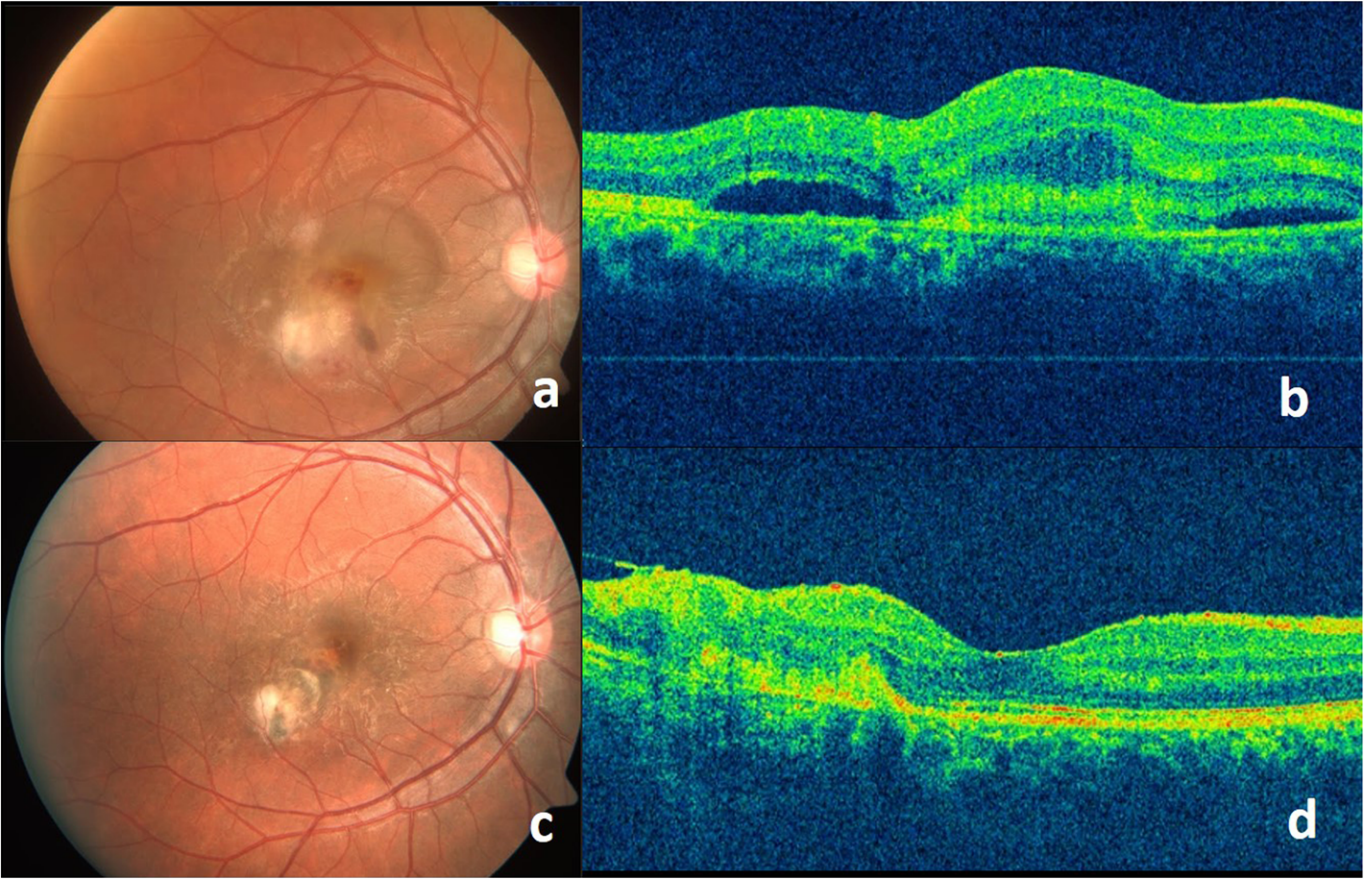
*At presentation*—**a** Color fundus photo of the right eye of case 1 shows a yellow-white active retinitis lesion (approximately 1 disc diameter, infero-temporal to fovea) adjacent to an old pigmented scar, a part of which is embedded in the active lesion. Coexisting subretinal hemorrhage was present at and inferior to the fovea along with macular thickening and subretinal fluid at the posterior pole. **b** Optical coherence tomography scan (*horizontal*) over the lesion shows an elevated foveal contour with increased retinal thickness, hyper-reflectivity, and pockets of subretinal fluid. *At 20 weeks of follow up*—**c** Color fundus picture shows healed, pigmented, and scarred lesion infero-temporal to fovea and **d** OCT scan over the lesion shows reduced retinal thickness, distorted architecture of retinal layers temporal to the fovea, reduced amount of subretinal fluid, and relative restoration of the foveal contour

A 15-year-old male patient presented with sudden onset blurring of vision in his right eye for 2 days and in the left eye for 2 years. His visual acuity at presentation in the right eye was 20/50, N18, and in the left eye 9/200, N36. Anterior segment findings were unremarkable. The right eye showed 1+ vitreous cells and a yellow-white active retinitis lesion (approximately 1 disc diameter, infero-temporal to fovea) adjacent to an old pigmented scar, a portion of which is embedded in the active lesion (Fig. 1). Coexisting subretinal hemorrhage was present at and inferior to the fovea along with macular thickening and subretinal fluid at the posterior pole. The left eye fundus showed disc pallor and a large (approximately 1.5 disc diameter in size), punched out, hyperpigmented scar at the macula. Fundus fluorescein angiography (FFA) and optical coherence tomography (OCT) (Fig. 1) confirmed the presence of a coexisting active lesion with classic choroidal neovascular membrane in the right eye. A diagnosis of recurrent *Toxoplasma* retinochoroiditis with active CNVM in the right eye and a healed *Toxoplasma* scar in the left eye was made. He was treated with an intravitreal injection of anti-vascular endothelial growth factor (anti-VEGF) (bevacizumab) along with oral anti-parasitic medication (320 mg trimethoprim and 1600 mg sulfamethoxazole—i.e., cotrimoxazole twice a day) along with anti-inflammatory medication (oral prednisone 60 mg/day). The visual acuity started improving within 1 week (right eye visual acuity—20/25 at 1 week with reduced subretinal fluid at macula). Cotrimoxazole was continued, and a dose of oral prednisolone was tapered over 1 month to 10 mg/day. Oral steroids were gradually tapered off while cotrimoxazole was discontinued after 2 weeks. At 20 weeks, the visual acuity was 20/20 with healing and scarring of the chorioretinal lesion (Fig. 1).

### Case 2

A 51-year-old female patient presented with diminution of vision in the right eye for 7 months. Visual acuity at presentation was 6/200 in the right eye and 20/20 in the left eye. Anterior segment examination was unremarkable. Fundus examination in the right eye showed minimal vitritis with a well-defined pigmented *Toxoplasma* scar and a yellowish-white necrotizing retinitis lesion adjacent to the scar with subretinal hemorrhage. FFA and OCT confirmed the presence of coexisting CNVM and active retinitis. She was treated with monthly injections of intravitreal anti-VEGF (bevacizumab) for 3 months along with an oral anti-*Toxoplasma* drug (cotrimoxazole) and a tapering dosage of oral corticosteroids for 6 weeks. After 4 months, her visual acuity improved to 20/200 in the right eye with healed chorioretinal scar with regression of CNVM.

### Case 3

A 32-year-old female presented with gradual diminution of vision in the left eye for 4 months. Visual acuity in the left eye was 3/200. Anterior segment examination of both eyes and fundus in the right eye was unremarkable. Fundus of the left eye had minimal vitritis with a yellowish-white retinitis lesion (at the posterior pole) and subretinal hemorrhage adjacent to a long-standing hyperpigmented scar (temporal to the fovea). FFA and OCT confirmed the presence of coexisting CNVM and active retinitis in the left eye. The left eye was treated with intravitreal injection of anti-VEGF (bevacizumab), oral anti-*Toxoplasma* drug (cotrimoxazole), and systemic corticosteroids. Intravitreal injection of bevacizumab was repeated at 1 month. The visual acuity improved to 20/100 and subretinal fluid regressed with healing and scarring of the retinitis lesion which remains stable at 2 months of follow-up.

### Discussion

In 1977, Willerson et al. [11] reported the first case of subretinal neovascularization in association with active *Toxoplasma* retinochoroiditis. The pathogenesis of CNVM during the active stage of *Toxoplasma* retinochoroiditis is thought to occur by a break in Bruch’s membrane and choriocapillaris [12] due to intense retinal inflammation. Because of this lesion, impeded retinal venous outflow may lead to active vasoproliferation and retinochoroidal vascular anastomosis [13]. Friable vascular channels may ultimately lead to formation of CNVM. Fundus fluorescein angiography (FFA) and optical coherence tomography (OCT) help in confirming the presence of coexisting neovascular membrane and active retinochoroiditis. Monnet et al. [14] described the OCT features of active toxoplasmosis as the presence of highly reflective intraretinal area corresponding with the area of retinitis, a thickened posterior hyaloid, and a shadow effect of the underlying choroidal tissue. CNVM during the healed stage generally occurs at the edge of the *Toxoplasma* scar [8]; while in the active stage, CNVM may be seen anywhere in the active retinitis lesion.

Options to treat CNVM secondary to resolved *Toxoplasma* retinitis include observation, corticosteroids, laser photocoagulation [15], photodynamic therapy (PDT) [16], submacular surgery [17], and intravitreal anti-VEGF agents [15, 18]. Management of CNVM in cases of healed toxoplasmosis with anti-VEGFs has been associated with reactivation of the retinochoroiditis lesion, so few authors do recommend concomitant use of oral anti-*Toxoplasma* treatment as prophylaxis [19]. However, if the CNVM coexists with the active stage of *Toxoplasma* retinochoroiditis, the combination therapy (anti-VEGF and anti-*Toxoplasma* treatment) becomes very important as the combined approach addresses both the active *Toxoplasma* lesion and the CNVM, thus achieving better anatomic and visual outcomes [20]. The anti-VEGFs are also effective in the management of subfoveal or juxtafoveal neovascular membrane as they also reduce the collateral tissue damage to neurosensory retina and choroid as is seen with PDT or laser photocoagulation or submacular surgery [20].

In conclusion, all three patients presented with coexisting CNVM with activation of retinochoroiditis. The diagnosis was based on clinical evaluation, FFA, and OCT. All three patients were promptly started on anti-*Toxoplasma* medical treatment along with intravitreal anti-VEGF injection guided by the clinical evaluation and the imaging findings. The lesions healed with regression of the neovascular membrane in all three patients with improvement in visual outcome and remained stable during a follow-up period ranging from 2 to 4 months.

## Abbreviations

anti-VEGF: anti-vascular endothelial growth factor
CNV: choroidal neovascularization
CNVM: choroidal neovascular membrane
FFA: fundus fluorescein angiography
OCT: optical coherence tomography
PDT: photodynamic therapy
